# Beware the intruder: Real time observation of infiltrated neutrophils and neutrophil—Microglia interaction during stroke *in vivo*

**DOI:** 10.1371/journal.pone.0193970

**Published:** 2018-03-15

**Authors:** Jens Neumann, Sophie Henneberg, Susanne von Kenne, Niklas Nolte, Andreas J. Müller, Burkhart Schraven, Michael W. Görtler, Klaus G. Reymann, Matthias Gunzer, Monika Riek-Burchardt

**Affiliations:** 1 Department of Neurology, Medical Faculty, Otto-von-Guericke University Magdeburg, Germany; 2 Institute for Experimental Immunology and Imaging, Medical Faculty, Essen, University Duisburg-Essen, Germany; 3 Institute for Molecular and Clinical Immunology, Medical Faculty, Otto-von-Guericke University Magdeburg, Germany; 4 Helmholtz Center for Infection Research, Braunschweig, Germany; 5 Project Group Neuropharmacology, Leibniz Institute for Neurobiology, Magdeburg, Germany; University Hospital-Eppendorf, GERMANY

## Abstract

Inflammation plays an important role in the pathogenesis of ischemic stroke including an acute and prolonged inflammatory process. The role of neutrophil granulocytes as first driver of the immune reaction from the blood site is under debate due to controversial findings. In bone marrow chimeric mice we were able to study the dynamics of tdTomato-expressing neutrophils and GFP-expressing microglia after photothrombosis using intravital two-photon microscopy. We demonstrate the infiltration of neutrophils into the brain parenchyma and confirm a long-lasting contact between neutrophils and microglia as well as an uptake of neutrophils by microglia clearing the brain from peripheral immune cells.

## Introduction

Stroke is the leading cause of lifetime morbidity and a major cause of death in industrialized countries. Inflammation plays an important role in the pathogenesis of ischemic stroke. This includes both an acute and a prolonged inflammatory process, characterized by rapid activation of resident microglia, production of proinflammatory mediators, and the early infiltration of neutrophils into the affected brain parenchyma followed by the recruitment of monocytes and T-cells [1]. Despite intensive investigation, the importance of microglia and neutrophils for stroke pathology is debated due to controversial findings [2]. In particular, it has been proposed that neutrophils less frequently cross the blood-brain-barrier (BBB) but instead are caught in the perivascular space and thus mainly remain located outside the brain parenchyma without having an effect on stroke outcome [3]. In contrast, we recently demonstrated the massive infiltration of LysM-eGFP^+^ -cells (that endogenously express eGFP in neutrophils) into the ischemic area beyond the perivascular space by intravital intracranial two-photon microscopy [4]. In addition, we could demonstrate an interaction of microglia and neutrophils as well as neutrophil phagocytosis by microglia *in vitro* [2] and a reaction of microglia by forming flat net-like membrane protrusions in response to infiltrated neutrophils *in vivo* [4].

However, LysM-eGFP^+^-mice are known to have not only neutrophils labeled with GFP, since also monocytes and other myeloid cells express low levels of GFP [5] in these mice. To overcome these limitations, we have now established a novel animal model in which neutrophils are exclusively labeled with tdTomato [6] and microglia/macrophages by EGFP [5]. By using this mouse model we could unequivocally demonstrate infiltrated neutrophil granulocytes within the ischemically affected brain parenchyma. In addition we were now able to show the interaction of microglia and neutrophils and the phagocytosis of neutrophil granulocytes by microglia *in vivo*.

## Materials and methods

### Transgenic mice

To selectively and specifically visualize neutrophils and microglia for intravital two-photon imaging we created bone marrow chimeras using the well-established method of lethal irradiation and bone marrow reconstitution [7]. In short, we used the novel genetic mouse model Catchup^IVM^ (C57BL/6^*Ly6g*(tm2621(Cre-tdTomato)Arte^ crossed to a tdTomato reporter mouse [6]) to visualize neutrophils. By transferring bone marrow of five Catchup^IVM^ mice into lethally irradiated (13gray for 4.5min) female CX3CR1-eGFP (n = 7), we generated mice with green fluorescent microglia (which are radio-resistant) and red fluorescent neutrophils from the donor bone marrow. An additional set of three Catchup^IVM^ bone marrows were transferred into three lethally irradiated C57BL6 recipients animals, to investigate the neutrophil infiltration into the brain parenchyma after cerebral ischemia without interfering microglial fluorescence. The transgenic mice strains were bred as heterozygotes. The animals were housed in groups under standard conditions (separately ventilated cages, light cycle 12:12 h including 2 h of twilight-phases, temperature 22–24 °C, humidity 55% ±10%). Food and water were available *ad libitum*. Experiments were performed in accordance with the European Communities Council Directives 86/609/EEC and 2010/63/EU and were formally approved by the responsible local authority (Landesverwaltungsamt für Kultur, Bauwesen und Verbraucherschutz, Referat Verbraucherschutz, Veterinärangelegenheiten (DZNE 42502-2-1244)). Experiments were reported according to ARRIVE (Animal Research: Reporting in Vivo Experiments) guidelines (S1 File).

### Surgical preparation

For the intracranial imaging we implanted cranial windows as described previously [8]. In short, mice were deeply anesthetized by inhalation of 4% Isoflurane initially in a box and further via a mask with 1.5–2.5% Isoflurane mixed with oxygen (250 ml/min). Body temperature was measured rectally and maintained at 37±0.5°C using a heating pad during all procedures (Temperature control, TSE Systems, Bad Homburg, Germany). The scalp was shaved with a small electrical razor and the mouse head was fixed in a stereotactic frame (SGM-4, Narishige, London, UK). The skin and the periosteum were removed from between the eyes to caudal of the ears. In between lamda and bregma a circular opening of was carved, moisturized with a drop of isotonic saline solution, and sealed with a cover slip (diameter = 7mm) which was fixed to the skull with tissue glue. The metal frame for the head holder was also glued on the scull and fixed additionally with the cover slip borders using dental cement.

### Two-photon intravital microscopy and photothrombosis

The two-photon intravital microscopy was performed 24 h after focal cerebral ischemia induced by photothrombosis [9]. Photothrombosis yields a distinct small cortical infarct. Hemorrhagic lesions usually identified by leakage of fluorescently tagged intravascular dextran have not been observed throughout the experiments. The mice were between 3 and 7 month old during these experiments. The anesthetized animals were fixed in the frame holder. For inducing the photothrombosis 50 μl of 0.1% Rose Bengal (Sigma-Aldrich, Darmstadt, Germany) were applied by retrobulbar injection and the brain was illuminated for 60 to 90 s with a laser pointer (532 nm wavelength, output <5 mW) through the cranial window. For two-photon microscopy we used an upright Zeiss (Jena, Germany) MP7 microscope equipped with a Coherent Chameleon laser (Goettingen, Germany) and a Zeiss 20x water-immersion objective (1.0 NA). Fluorescence was detected at 500–550 nm for GFP and 565–610 nm for tdTomato by non-descanned detectors following excitation with 920 nm. During the *in vivo* microscopy the laser was used with a power between 7 and 13% depending on imaging depth.

To assure an appropriate depth of imaging, we performed z-stacks using SHG-signal to identify the meninges and subsequently established the imaging depth below the meninges as described before [4]. Thus, CX_3_CR_1_-eGFP positive flat shaped vascular associated macrophages were not found in the region of interest. After two-photon imaging the mice were sacrificed by an overdose of isoflorane.

### Histology

The fluorescence staining with fluorescein coupled *solanum tuberosum* lectin was performed in 50 μm thick frozen sections from bone marrow chimeric mice (C57BL6 reconstituted with bone marrow from Catchup^IVM^ mice). The brains were fixed with 4% paraformaldehyde 24 h after photothrombosis by transcardial perfusion. Microscopic pictures were done with a Leica SP8 (Wetzlar, Germany) confocal microscope (objective: 20x water-immersion, excitation of FITC: 488 nm, Catchup^IVM^: 561 nm). IMARIS (Bitplane, Zurich) was used for preparing the movies and images.

### Statistical analysis

IMARIS (Bitplane, Zurich) was used for automated tracking (velocity) and sphericity measurements of neutrophils. Statistical analysis were performed using GraphPadPrism (LA Jolla, USA). For comparison of two groups unpaired two-sided Student t-tests were applied. A value of p<0.05 was considered statistically significant.

## Results

### Neutrophils infiltrate the brain parenchyma after focal cortical ischemia

**Fig 1:**
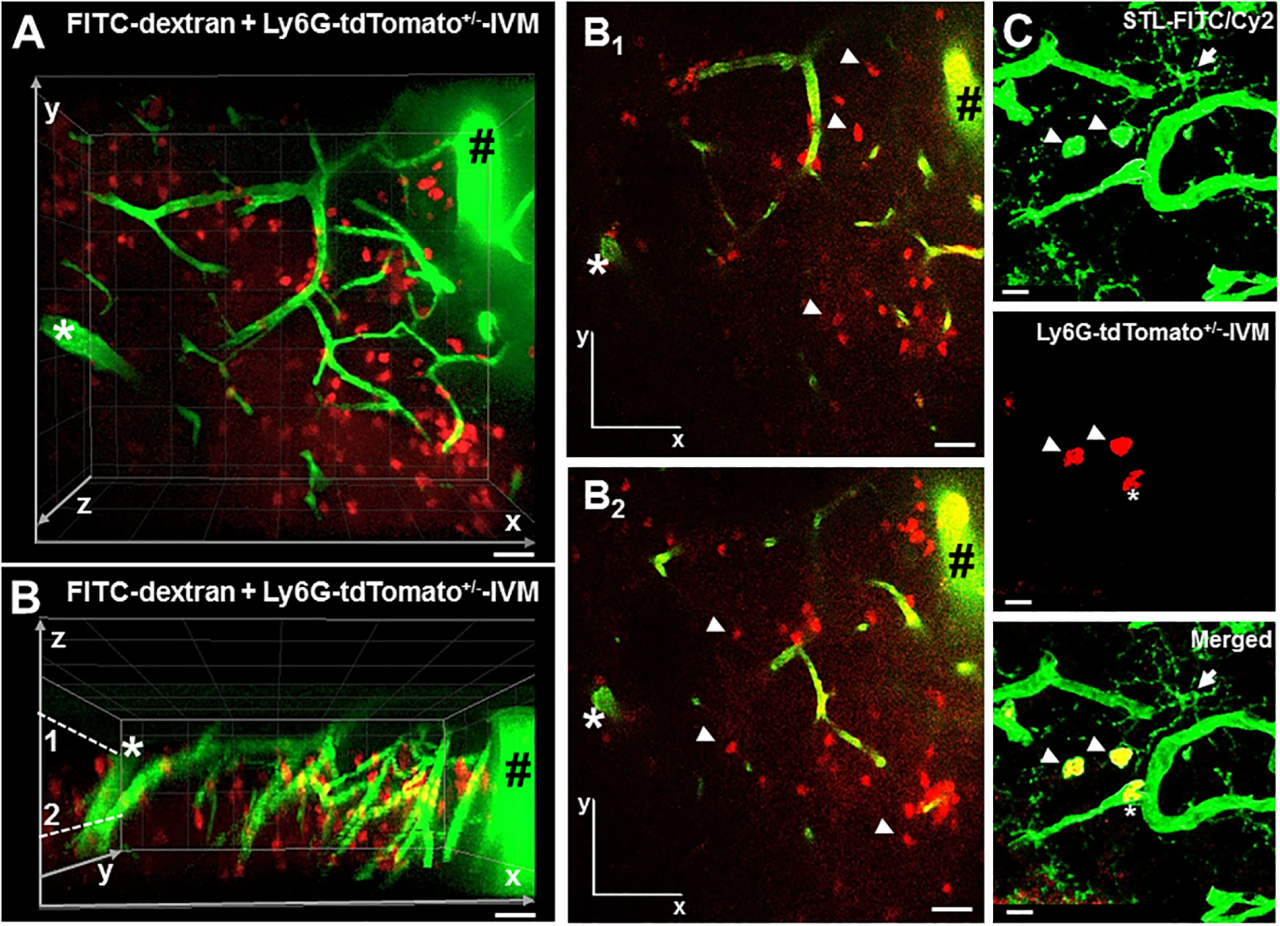
Neutrophils infiltrate the ischemic affected brain parenchyma after photothrombosis. **A** C57BL/6 animals were lethally irradiated and reconstituted with bone marrow from Catchup^IVM^ mice. 8 weeks after recovery mice were subjected to photothrombosis and the affected brain areas investigated by intravital 2-photon microscopy. Shown is a 3D reconstruction (x-y orientation) of infiltrated tdTomato^+^ neutrophils (red, white arrows) within the infarct zone as well as FITC-dextran^+^ capillaries (green). **B** 3D reconstruction of **A** showing the depth (z orientation) of neutrophil infiltration. **B**_**1**_ and **B**_**2**_ displaying 2D transversal sections in x-y orientation in different depth of ***B***. The arrowheads in **B**_**1**_ and **B**_**2**_ showing infiltrated neutrophils in the parenchyma apart the capillaries. **A, B, B**_**1**_ **and B**_**2**_ The black rhombus and the white asterisk show two cortex penetrating vessels with the capillary bed in between indicating an appropriate depth of imaging. **C** Staining of a 50 μm thick section displaying infiltrated tdTomato^+^ neutrophils (red, arrowheads) within the parenchyma and a neutrophil in a capillary (asterisk) as well as showing neutrophils, capillaries and a microglia (arrow) in green by using FITC-labeled *solanum tuberosum* (Potato) lectin. Scale bars: A, B, B_1_, B_2_: 10 μm and C 5 μm.

Due to the superficial localization of the photothrombotic infarct, this approach is ideal for intracranial microscopy regarding the infiltration of blood cells into the brain cortex and interaction between cells using fluorescent protein reporter mouse models. For investigating the post-ischemic neutrophil invasion, we generated bone marrow chimeras of wild type (C57BL/6) hosts reconstituted with bone marrow from Catchup^IVM^ mice. Twenty four hours after induction of photothrombosis, we detected tdTomato-positive neutrophils in the capillary bed between cortical penetrating vessels of the affected brain parenchyma (Fig 1A, 1B and 1C). These cells were motile (S1 Movie) and mainly located beyond the perivascular space within the brain parenchyma (Fig 1A, 1B, 1B1 and 1B2). To underpin the intravital *in vivo* observations, we performed histological examination to determine the localization of neutrophils relative to the capillaries by staining with FITC-coupled *solanum tuberosum* (Potato) lectin (STL) to visualize endothelial cells in particular as well as microglia and neutrophils. This investigation reveals neutrophil localization in the parenchyma without being caught in the perivascular space (Fig 1C and S1 Fig).

Thus, using the Catchup^IVM^ model we could confirm unequivocally living and motile neutrophils intraparechymal in ischemically injured brain.

### Microglia cells react to passing neutrophils, interact with and engulf them

**Fig 2:**
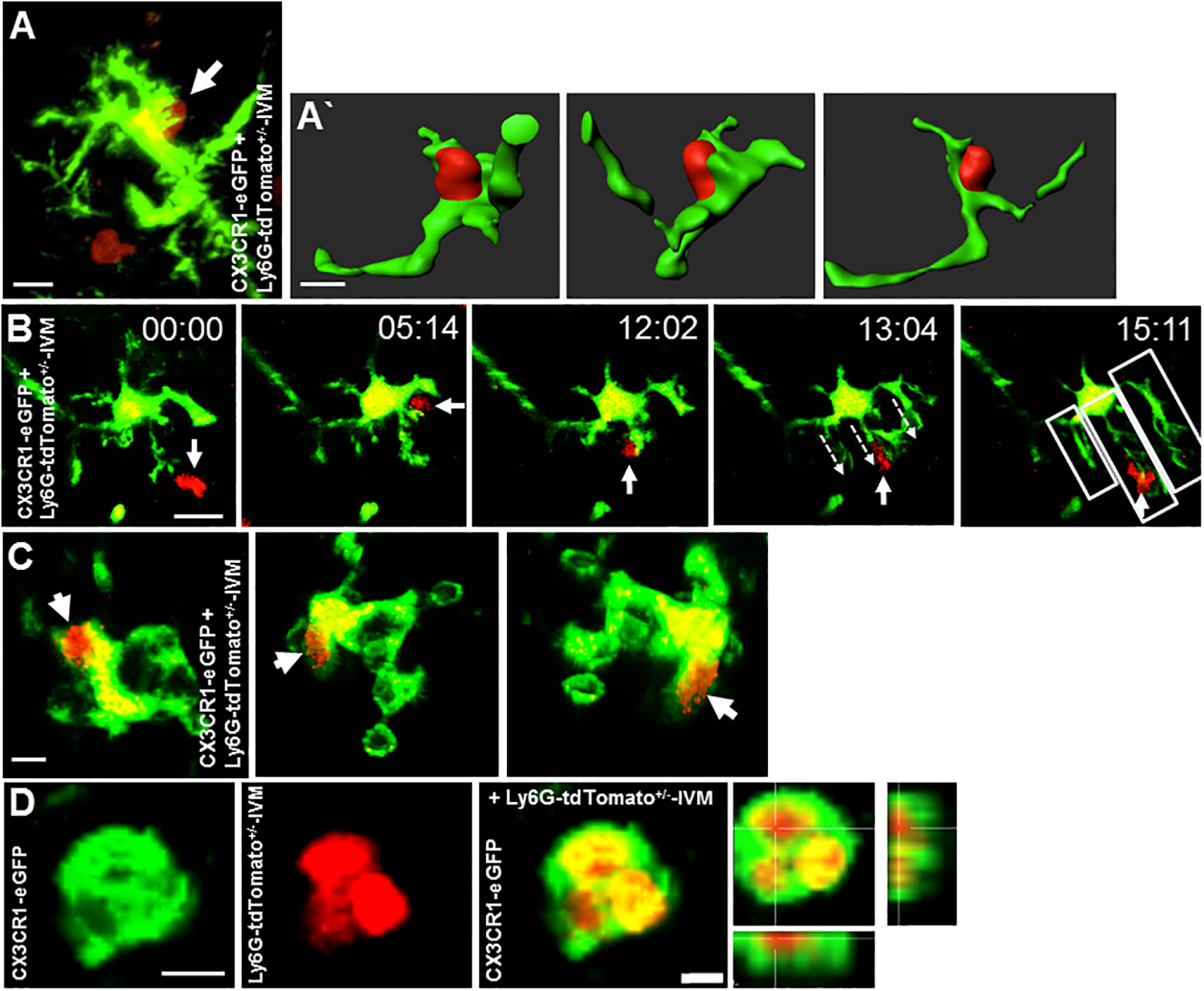
Interaction of microglia and neutrophil granulocytes 24 h after photothrombosis. **A** The panel demonstrates a direct physical contact which is reconstructed and displayed in different angles in **A´**. The time series in **B** show the reaction of a microglia on a passing neutrophil granulocytes by extension of the protrusions towards the neutrophil granulocytes. The arrows mark the neutrophil granulocytes, the dotted arrows show the direction of the protrusions and the boxes in the last picture highlight the 3 extended protrusions. The panel **C** demonstrates an intracellular located neutrophil granulocytes (Catchup^IVM^) in a microglia (CX_3_CR1-eGFP^+^) from different angles. The pictures in **D** show the uptake of multiple neutrophil granulocytes by a microglia. Scale bars: A, C 10 μm, B, B`, D, E 5 μm.

Having confirmed the infiltration of neutrophils into the ischemic brain parenchyma, we next sought to investigate the interaction between invaded neutrophils and microglia. Our previous work demonstrated the interaction of microglia and neutrophils *in vitro* [2] and a reaction of microglia towards infiltrated LysM-eGFP^+^ cells *in vivo* [4], by flattening their processes, but due to eGFP-labeling of both microglia and neutrophils in the mouse model used in this work. An unequivocal proof of the interaction between neutrophils and microglia *in vivo* was not possible at the time. Using our new chimeric mouse model containing eGFP^+^ microglia as well as tdTomato^+^ neutrophils, we found intraparenchymal neutrophils which showed extensive cell-cell contacts with microglia (Fig 2A and 2A`) after photothrombosis. In some cases, these contacts lasted several minutes (S2 Movie). Furthermore, we observed microglia, which reacted by extending their processes towards passing neutrophils (Fig 2B and S3 Movie). Finally, we could detect activated, ameboid shaped microglia in the parenchyma, which had clearly internalized one or several infiltrated neutrophils (Fig 2C and 2D). In summary, we succeeded to visualize neutrophils internalized by microglia in two mice, and in four mice we detected multiple neutrophil-microglia interactions. These observations confirm specific interactions of microglia and live neutrophils in ischemia-affected brains *in vivo*.

### Neutrophil dynamic behavior in ischemic brain

**Fig 3:**
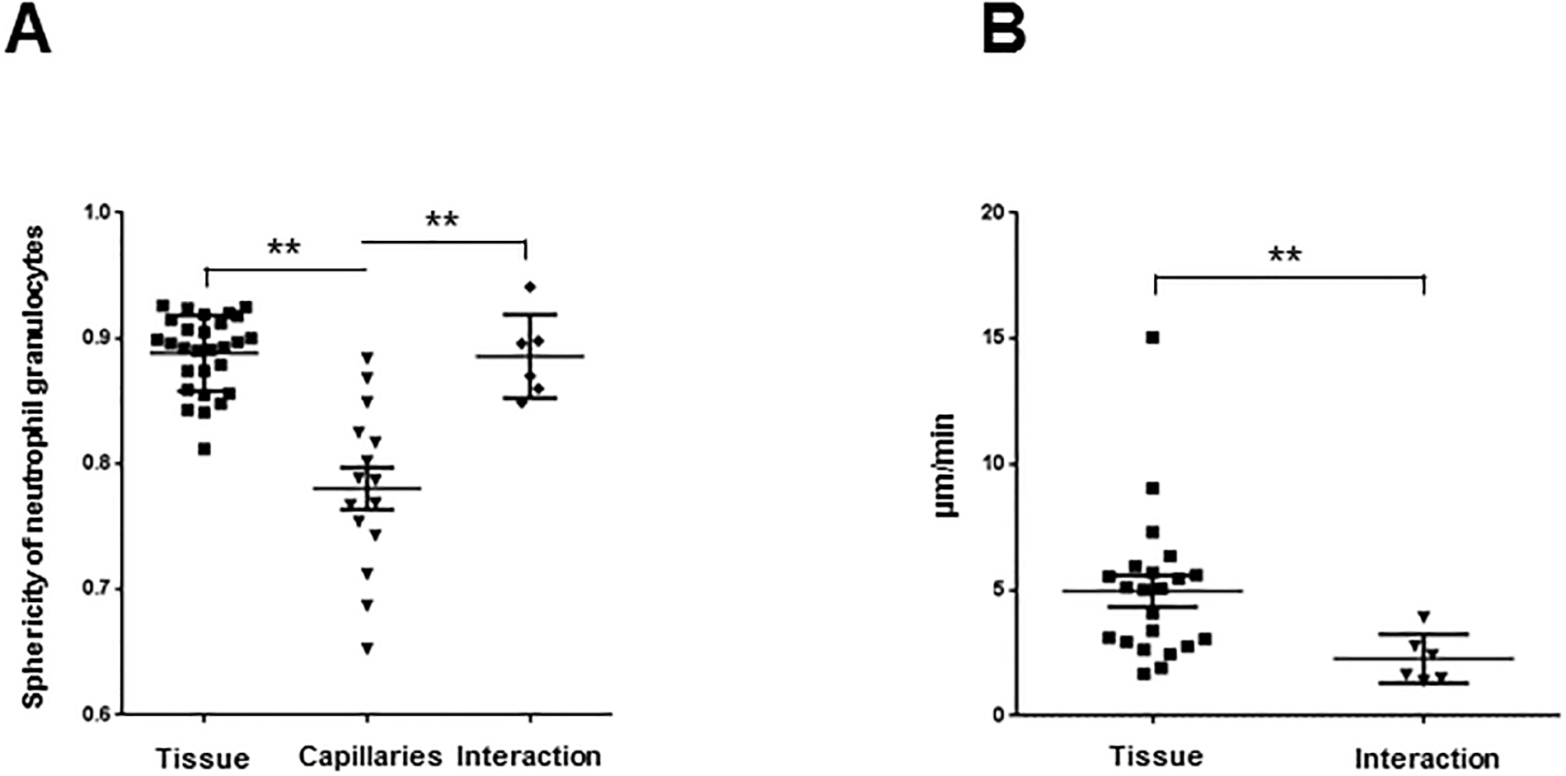
The automatically analysis of the different neutrophil granulocytes conditions. **A** Quantification of the sphericity of neutrophil granulocytes inside the capillaries (15 cells), in the tissue (28 cells) and in contact with microglia (6 cells) (**p<0.01 capillaries vs. tissue and interaction, n = 4 mice). ***B*** Quantification of neutrophil granulocytes velocity in the tissue (22 cells) and in contact with microglia (6 cells) (**p<0.01 tissue vs. interaction, n = 4 mice).

After onset of cerebral ischemia, neutrophils showed different activities. Neutrophils either (i) plugged the capillaries or squeezed (S4 Movie) through them. (ii) Moving cells inside the ischemic parenchyma were migratory unless they got (iii) in contact with microglia. In order to quantify these different behaviors, we first automatically analysed the sphericity of neutrophils. This enabled us to classify neutrophils inside the parenchyma as cells exhibiting almost perfectly round shapes in contrast to neutrophils inside the capillaries with much more elongated structures (Fig 3A). In addition, when we analysed the velocity (Fig 3B) of neutrophils in the parenchyma, we found significant differences between freely moving neutrophils in the parenchyma, which were highly motile, and nearly immotile neutrophils that were in contact with microglia. This further underlined a distinct neutrophil-microglia interaction behavior.

Taken together, we were able to track and automatically quantify neutrophil infiltration into, and neutrophil-microglia interactions within the brain parenchyma after cerebral ischemia.

## Discussion

This study was designed to address questions related to the debate of whether or not neutrophils infiltrate the brain parenchyma after ischemic injury and what their fate thereafter might be.

Neutrophil infiltration after cerebral ischemia is still debated, and knowledge on the interaction between infiltrated neutrophils and activated microglia is therefore scarce. Enzmann et al. [3] showed that neutrophils do not cross the perivascular space of cerebral vessels in a substantial number after transient ischemia. In contrast, we and others previously demonstrated that Lys-M-eGFP^+^-cells infiltrate the brain parenchyma, are motile beyond the cerebral vessels and within the parenchyma [4, 10]. With our new animal model we were now able to demonstrate that neutrophils clearly belong to the type of cells infiltrating the brain.

Further studies are needed to quantify the amount of neutrophils relative to other infiltrating peripheral immune cells and the response of microglia towards this insult. This cannot be achieved by simple flow cytometric counting or static histology, as both, the local context and the involved cellular dynamics are essential to the process. Theses parameters can only be obtained by intravital imaging.

Interactions between microglia and neutrophils could last several minutes. In addition, we detected microglia reacting on passing neutrophils as well as microglia with single or multiple ingested neutrophils. The molecular pathways involved in such diverse interactions are enigmatic at present and require the further refinement of the animal models involved, e.g. by defining the protein composition of the interacting cells to search for potential interaction partners. We have shown previously, that RGD peptides or GlcNAC are able to inhibit microglia-neutrophil interactions *in vitro* [2]. This suggests that integrin-based interaction and/or lectins are involved in the process. Future studies should therefore aim at deleting such proteins specifically from either microglia or neutrophils *in vivo* and then test the influence of this on the development of experimental stroke. Since we could show in brain slice models that the interference with microglia-neutrophil interaction is blocking a neuroprotective role of microglia, it is conceivable, that a generic animal model of such a blockade would lead to increased damage of experimental stroke. Showing such connections might ultimately pave the way to novel treatment options of stroke that aim at neuroprotection via the modulation of microglia-neutrophil interactions.

## Conclusions

In summary, with our new chimeric mouse model we demonstrate the infiltration of neutrophils in experimental superficial cortical stroke by photothrombosis and interaction of neutrophils and microglia as well as phagocytosis of neutrophil granulocytes by the microglia. Whether this has a role for limiting neutrophil-mediated brain pathology remains an important question for future investigation.

## Supporting information

S1 Fig Localization of infiltrated neutrophils within the parenchyma. Staining of a 50 μm thick section displaying tdTomato^+^ neutrophils (numbered) and FITC-labeled *solanum tuberosum* (Potato) lectin (STL) positive neutrophils and capillaries. The section has been recorded as z-stack. Since FITC-coupled STL labels capillaries and neutrophils as well, we looked at every neutrophil individually to determine the localization in relation to the capillary. The z-stack data ***(1*, *2*, *3)*** of each neutrophil revealed, that the neutrophils ***1*** and ***2*** are located in the parenchyma whereas neutrophil ***3*** is found inside the capillary. Scale bars: 5 μm.
(TIF)

S1 Movie Neutrophil granulocytes infiltrate the ischemic affected brain parenchyma after photothrombosis. The cerebral capillaries were visualized by retrobulbar injection of FITC/dextrane (70kDa) in green and the neutrophil granulocytes are shown in magenta (Catchup^IVM^). The tracks mark neutrophil movement within the brain parenchyma. Z-stacks (15 images with a distance of 1.5μm) were obtained in 30s intervals for a period of 16min. Scale bar: 50 μm.
(AVI)

S2 Movie Physical contact between microglia and neutrophil granulocytes 24 h after photothrombosis. The movie, done with 2-photon microscopy, indicates a green microglia (CX_3_CR1-eGFP^+^) in close contact with neutrophil granulocytes in magenta (Catchup^IVM^) over minutes. The white color, appearing during microglia-neutrophil interaction, indicates the contact zones between both cell types (calculated by Imaris). Images were done in bone marrow chimeras (C57Bl/6 animals as recipients and Catchup^IVM^ as donor). Images were optained in 5 s intervals for a period of 2 min.
(MOV)

S3 Movie Recognition of neutrophil granulocytes by microglia 24 h after photothrombosis. Two-photon microscopy was used in bone marrow chimeras to investigate the microglia-neutrophil interactions after experimental stroke *in vivo*. After passing the microglia (CX_3_CR1-eGFP^+^-eGFP, green) by the neutrophil granulocyte (Catchup^IVM^, magenta), the microglia reacts by extending its membrane protrusions towards the neutrophil granulocyte. Z-stacks (15 images, interval 1.5 μm) were obtained in 30 s intervals for a period of 16 min.
(MOV)

S4 Movie Neutrophil plugged the capillaries after photothrombosis. Neutrophil granulocytes in red (Catchup^IVM^) plugged the capillaries or squeezed slowly through them. The cerebral capillaries were visualized by retrobulbar injection of FITC/dextrane (70kDa) in green. Images were obtained in 7 frames per minute for 11 min.
(MP4)

S1 File ARRIVE guidelines checklist. (DOCX)

## References

[1] Chamorro A, Dirnagl U, Urra X, Planas AM. Neuroprotection in acute stroke: targeting excitotoxicity, oxidative and nitrosative stress, and inflammation. Lancet Neurol. 2016;15(8):869–81. doi: 10.1016/S1474-4422(16)00114-9 27180033 10.1016/S1474-4422(16)00114-9

[2] Neumann J, Sauerzweig S, Ronicke R, Gunzer F, Dinkel K, Ullrich O, et al Microglia cells protect neurons by direct engulfment of invading neutrophil granulocytes: a new mechanism of CNS immune privilege. J Neurosci. 2008;28(23):5965–75. doi: 10.1523/JNEUROSCI.0060-08.2008 18524901 10.1523/JNEUROSCI.0060-08.2008 PMC6670327

[3] Enzmann G, Mysiorek C, Gorina R, Cheng YJ, Ghavampour S, Hannocks MJ, et al The neurovascular unit as a selective barrier to polymorphonuclear granulocyte (PMN) infiltration into the brain after ischemic injury. Acta Neuropathol. 2013;125(3):395–412. doi: 10.1007/s00401-012-1076-3 23269317 10.1007/s00401-012-1076-3 PMC3578720

[4] Neumann J, Riek-Burchardt M, Herz J, Doeppner TR, Konig R, Hutten H, et al Very-late-antigen-4 (VLA-4)-mediated brain invasion by neutrophils leads to interactions with microglia, increased ischemic injury and impaired behavior in experimental stroke. Acta Neuropathol. 2015;129(2):259–77. doi: 10.1007/s00401-014-1355-2 25391494 10.1007/s00401-014-1355-2

[5] Jung S, Aliberti J, Graemmel P, Sunshine MJ, Kreutzberg GW, Sher A, et al Analysis of fractalkine receptor CX(3)CR1 function by targeted deletion and green fluorescent protein reporter gene insertion. Mol Cell Biol. 2000;20(11):4106–14. 10805752 10.1128/mcb.20.11.4106-4114.2000 PMC85780

[6] Hasenberg A, Hasenberg M, Mann L, Neumann F, Borkenstein L, Stecher M, et al Catchup: a mouse model for imaging-based tracking and modulation of neutrophil granulocytes. Nat Methods. 2015;12(5):445–52. doi: 10.1038/nmeth.3322 25775045 10.1038/nmeth.3322

[7] Turrin NP, Plante MM, Lessard M, Rivest S. Irradiation does not compromise or exacerbate the innate immune response in the brains of mice that were transplanted with bone marrow stem cells. Stem Cells. 2007;25(12):3165–72. doi: 10.1634/stemcells.2007-0508 17761757 10.1634/stemcells.2007-0508

[8] Shih AY, Driscoll JD, Drew PJ, Nishimura N, Schaffer CB, Kleinfeld D. Two-photon microscopy as a tool to study blood flow and neurovascular coupling in the rodent brain. J Cereb Blood Flow Metab. 2012;32(7):1277–309. doi: 10.1038/jcbfm.2011.196 22293983 10.1038/jcbfm.2011.196 PMC3390800

[9] Lee JK, Park MS, Kim YS, Moon KS, Joo SP, Kim TS, et al Photochemically induced cerebral ischemia in a mouse model. Surg Neurol. 2007;67(6):620–5; discussion 5. doi: 10.1016/j.surneu.2006.08.077 17512331 10.1016/j.surneu.2006.08.077

[10] Liesz A, Zhou W, Mracsko E, Karcher S, Bauer H, Schwarting S, et al Inhibition of lymphocyte trafficking shields the brain against deleterious neuroinflammation after stroke. Brain. 2011;134(Pt 3):704–20. doi: 10.1093/brain/awr008 21354973 10.1093/brain/awr008

