# Supplementary figures and images for: Beware the intruder: Real time observation of infiltrated neutrophils and neutrophil—Microglia interaction during stroke in vivo
Source: PLoS One. 2018 Mar 15;13(3):e0193970. doi: 10.1371/journal.pone.0193970 (PMC5854356; doi:10.1371/journal.pone.0193970)

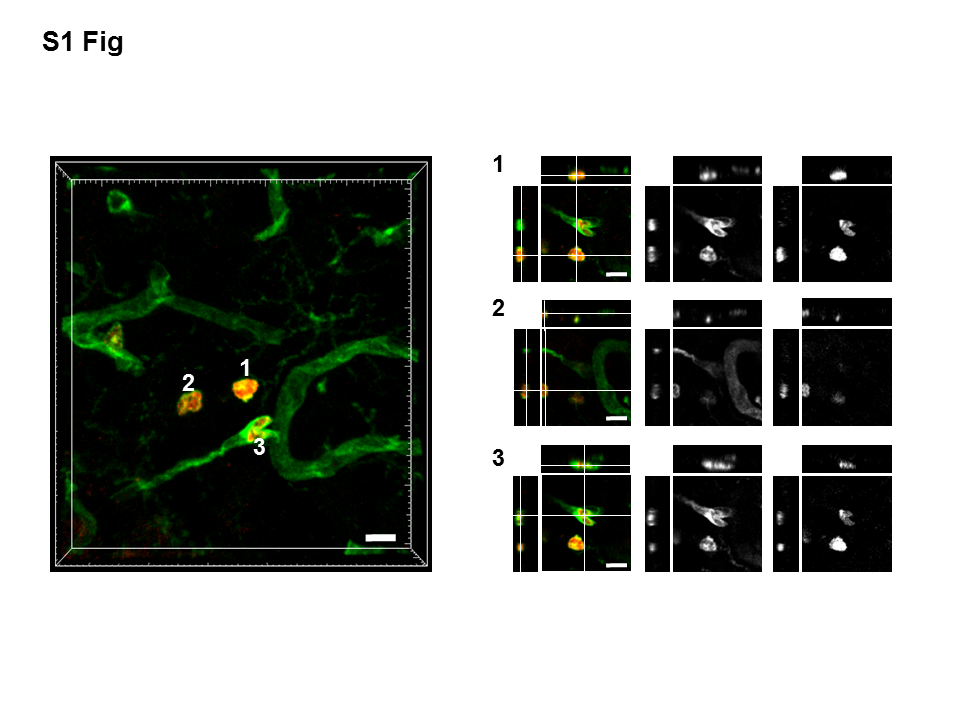

Supplement: S1 Fig — Staining of a 50 μm thick section displaying tdTomato+ neutrophils (numbered) and FITC-labeled solanum tuberosum (Potato) lectin (STL) positive neutrophils and capillaries. The section has been recorded as z-stack. Since FITC-coupled STL labels capillaries and neutrophils as well, we looked at every neutrophil individually to determine the localization in relation to the capillary. The z-stack data (1, 2, 3) of each neutrophil revealed, that the neutrophils 1 and 2 are located in the parenchyma whereas neutrophil 3 is found inside the capillary. Scale bars: 5 μm. (TIF) [file pone.0193970.s001.tif]
